# Warning against Critical Slopes in Agriculture: Comprehension of Targeted Safety Signs in a Group of Machinery Operators in Italy

**DOI:** 10.3390/ijerph16040611

**Published:** 2019-02-20

**Authors:** Lucia Vigoroso, Federica Caffaro, Eugenio Cavallo

**Affiliations:** Institute for Agricultural and Earthmoving Machines (IMAMOTER), National Research Council of Italy (CNR), Strada delle Cacce, 73–10135 Torino, Italy; l.vigoroso@ima.to.cnr.it (L.V.); eugenio.cavallo@cnr.it (E.C.)

**Keywords:** agriculture, injury risk, occupational safety, road sign comprehension, slope angle, warning sign

## Abstract

Steep slopes are the main cause of rollover incidents in agriculture. Targeted safety signs have been developed to warn machinery operators against risky slopes. However, machinery user’s manuals and road signs report information regarding slope steepness in two different ways, by using the tilt angle in degrees and the slope percentage, respectively. In this study, we investigated the comprehension of safety signs depicting critical slopes, either in degrees or as percent values in a group of Italian agricultural machinery operators while considering the possible influence of previous experience with agricultural machinery, previous incidents, and on-farm occupation. Eighteen tractor and self-propelled machinery operators were administered graphical representations of seven slope angles in a randomized order and then were asked to estimate the slope steepness as both a tilt angle and a slope percentage. The participants tended to overestimate slope steepness in degrees, whereas the opposite was true for percentages. Farmers who were previously involved in a machinery-related incident were more accurate in their estimates. The present results raise some considerations regarding the need to redesign safety communication and to promote targeted training interventions.

## 1. Introduction

Agriculture is one of the most dangerous industries, with an incident rate that is nearly triple that of other sectors, both in the United States and in the European Union [1,2]. Nearly 80% of all incidents are caused by farm machinery, and tractors are involved in most of the fatalities [3,4], with rollovers predominating [5]. The National Institute of Occupational Safety and Health (NIOSH) reported approximately 130 tractor rollover incidents per year in the United States [6]; Darçın and Darçın [7] reported that, in Bilecik, Turkey, 58 out of 80 tractor fatalities between 2005 and 2015 were due to rollovers, whereas Kogler et al. [8] and Pessina and Facchinetti [9] showed that tractor rollover resulted in many fatal injuries in Austria and in Italy in 2015 and 2017, respectively. In Italy, in particular, approximately 90 of 120 fatalities involving tractors in 2016 were fatal rollovers [10]. In addition to tractors, many other off-road self-propelled machines with a ride-on operator (driver) adopted in farming, such as harvesters [11] or handling machinery [12], or farm-like (lawnmowers, All Terrain Vehicles (ATV) and Side-by-Side) [13] operations are exposed to the risk of rolling or tipping over. According to Abubakar et al. [5] driving a vehicle downhill, crossing a steep slope, and climbing uphill represent the main reasons for machine rollovers. Previous studies showed also that agricultural machinery operators often disregard the steepness of terrain [13,14,15] and they tend to be inaccurate when estimating slopes [5,14]. This may lead to driving in hazardous conditions, thereby increasing the risk of a rollover incident [16].

To ensure the health and safety of workers, a five-step hierarchy of controls should be applied to the design of machinery and equipment [17,18]. The first three steps, i.e., elimination (step 1), substitution (step 2) and engineering controls (step 3), in particular, are the preferable methods to avoid a worker’s exposure to dangerous situations, since they remove the hazards at the source. Regarding machinery rollover, some studies have commented on possible solutions (step 1 and step 2) to improve tractor stability [19]. However, it is difficult to completely eliminate the hazard and to guarantee the comprehensive stability of the vehicle, since the risk of overturning is the result of the interaction between different factors, including the slope steepness [5,20]. Therefore, different technical protective measures can be adopted to prevent injuries (step 3) in the case of a rollover: the combined use of a Roll-Over Protective Structures (ROPS) and a seatbelt is the most effective way to prevent fatal injuries during rollover incidents [6,21,22,23]. The effectiveness of such features has been investigated and recognized for many years, and they have been required to be fitted to tractors since the ’80s in the markets of developed countries [24,25,26]. Retrofitting programmes to implement ROPS on tractors that were originally sold without a ROPS and a seatbelt have been recently implemented both in the United States and in the European Union to extend the benefits of these devices to a larger number of units [21,27]. Although “eliminating hazards is the most effective way to make the workplace safer […] this is not always practical” [28]. Thus, there is a need to focus on the work activity and to push towards the correct use of safety tools. To that end, the fourth step of the hierarchy of controls, i.e., administrative controls, includes training and the installation of signs, warning labels, and pictograms to inform users about the residual risks that could not be eliminated either by design or by adopting technical solutions [16,17].

The comprehension of safety signs in agriculture has been previously investigated with regard to pesticides [29,30], machinery-related risks [31], and the use of Personal Protective Equipment [32], and the research has often highlighted poor comprehensibility and a positive relation with the expertise of the target audience [33,34]. For instance, Caffaro, Schmidt, Murphy, and Cavallo [34] showed that years of experience with agricultural machinery significantly increased users’ comprehension of the meaning of safety pictograms that were affixed to agricultural machinery.

However, the comprehensibility of safety signs on slopes has not been investigated yet. Increasing knowledge about this issue is even more relevant if we consider that international standards and user manuals for safe operation of machinery do not adopt a unique graphical solution to depict critical steepness. On the one hand, the international standard ISO 16231-2 [35] for the assessment of stability of self-propelled agricultural machinery defines the maximum slope on which a machine is intended to work as a percentage; on the other hand, in OECD Code 6 [36], for the testing of front-mounted ROPS fitted on tractors and in the American National Standard ANSI/ROHVA 1-2016 [37] for determining the maximum steepness to operate ATVs, the slopes are described in degrees. Furthermore, the user manuals of different agricultural machinery display information regarding the maximum slope to operate a vehicle as a percentage [38,39], whereas the safety labels that were affixed to machinery give the same information in degrees [21]. Finally, in the Vienna Convention on Road Signs and Signals [40], the well-known road sign warning against a dangerous slope depicts the slope in terms of a percent value.

Based on these previous considerations, the present study investigated which kind of graphical representation of slope steepness, i.e., the tilt angle in degrees or a slope percentage, in a safety sign that was administered to a group of agricultural machinery operators was more comprehensible.

Furthermore, the study investigated the role that is played by the driving experience of agricultural machinery, the on-farm occupation, and the previous history of incidents in the comprehension of the safety sign. Experience with machinery was considered because of its known significant influence on the comprehension of safety signs [41], whereas we were interested in exploring the role of the other two variables based on previous evidence regarding their relevance in the field of risk perception and exposure in agriculture.

Concerning on-farm occupation, Day et al. [42] observed that farmworkers and contractors reported increased injury risk when compared with farmers. Whereas farmers spent longer daily working hours on farms [43], farmworkers and contractors interacted with many hazardous tools and equipment that could increase their risk exposure [42]. Concerning operators’ previous history of incidents, contrasting results are reported in the literature. McLaughlin and Sprufera [44] showed that operators who are involved at least once in an incident without negative consequences feel more confident in their interaction with machinery, thereby increasing their exposure to risky situations; on the other hand, in a study that was conducted by Caffaro et al. [45], operators who survived incidents with farm machinery developed a positive attitude towards safety and increased risk perception.

By analysing which kind of graphical representation of slopes (i.e., degrees or percentages) on safety signs was better comprehended and which variables affected the comprehension, this study aimed to provide useful suggestions in terms of the re-design of safety signs on slopes and/or targeted training activities to improve safety communication.

## 2. Materials and Methods

### 2.1. Participants

The investigation was performed in Italy because of the high number of machinery rollover-related fatalities [46] in this country. Eighteen Italian operators of tractors and self-propelled agricultural machinery (17 males, one female) from Northern Italy participated in the study. The participants were recruited from different agricultural holdings near Torino, Piedmont region, North western Italy. To obtain more comparable data, only participants who were familiar with agricultural machinery, i.e., with at least five years of operating experience (cutoff for experts, as in Kumar et al. [47]), were involved in the study. The study was conducted in accordance with the Declaration of Helsinki and the protocol was approved by the Research Advisory Group of the Institute for Agricultural and Earthmoving Machines (IMAMOTER) of the National Research Council of Italy (CNR) on February 16, 2018.

### 2.2. Material and Procedure

Seven different levels of slope steepness were chosen for the investigation. Based on the study that was conducted by Abubakar et al. [5] and Murphy et al. [19] regarding the stability limits for different farm tractors, five tilt angles were chosen: 5°, 10°, 15°, 20°, and 25°, which corresponded to slope percentages of 9%, 18%, 27%, 36%, and 47%, respectively. Two other angles were considered, as follows: 38°, corresponding to 78%, which is the angle of inclination that is used for tractors’ preliminary lateral stability test conducted in accordance with OECD Code 6 [36], and 45°, with a corresponding percentage of 100%.

**Figure 1:**
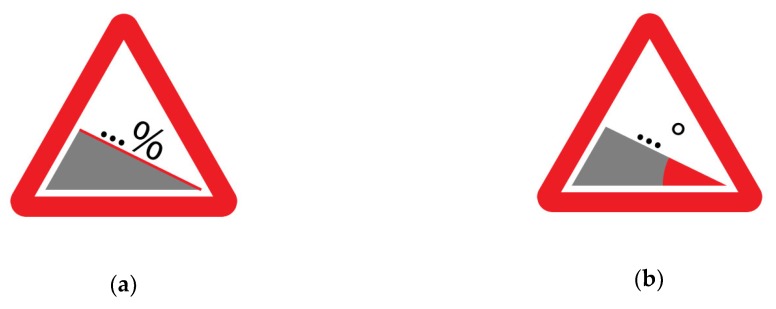
Example of the two versions of the safety sign submitted to users’ attention: (**a**) 36% slope and its corresponding (**b**) 20° angle.

To investigate the above-indicated angles, we referred to a sign that warns against a steep slope, as designed in the Vienna Convention on Road Signs [40] and to the study that was conducted by Paniati [48], in which different alternatives of existing road signs were tested. The safety sign that was considered in this study is characterized by two elements: a black triangle that represents the slope and a slope percentage value above it. For the estimation of the slope steepness, two versions of the sign were designed with two different graphical indications to focus users’ attention on the unit of measurement that had to be reported. For the estimation as a percentage, the element that represented the slope was underlined using a red line, with a percentage symbol (%) that was above this line. For the estimation in degrees, a small coloured arch that was used when dimensioning angles in a technical drawing was depicted to highlight the angle considered, and the degree symbol (°) replaced the percentage symbol adopted for the other version (see Figure 1). A total of fourteen signs (i.e., seven slopes to be expressed in degrees and seven as percentages) printed on 210 × 297 mm paper were presented in a random order to the participants. Participants were asked to indicate the degree of the tilt angle that was highlighted in red and the percentage of inclination of the red line with respect to the grey triangle’s base. When a sign reporting the percentage symbol (%) was shown to the participant, the following instructions were given: “In your opinion, what is the slope percentage of the red line compared to the grey triangle’s base?” Similarly, when a sign reporting the degree symbol (°) was shown, the participant was asked the following: “In your opinion, how many degrees is the red coloured angle in the picture?”

The participants were not informed that each slope was shown to them twice, once with the percentage symbol (%) and once with the degree symbol (°), so as not to affect their answers.

Participants were individually shown the signs. All of the answers were verbally reported [49,50] and the interviewer recorded them. The instrument was pilot-tested before being used in the present investigation. A sociodemographic questionnaire assessing participants’ occupation, years of experience with agricultural machinery, and history of previous incidents with agricultural machinery (dichotomous item, 0 = never involved and 1 = involved, as previously coded by Glasscock et al. [51]) closed the session.

#### Data Analysis

The answers that were reported by participants were classified according to the study conducted by Cavallo et al. [21], i.e., considering the limit for an acceptable estimation at ±2.5 degrees of the actual angle. In the absence of similar studies regarding percentages, the ±2.5 degrees limit was converted into a percentage value to use the same indicators to evaluate the users’ estimation. Thus, the limit for the acceptable estimation of slopes expressed as percentages was set at ±4%. For descriptive statistics, the underestimations were coded as −1, correct answers were coded as 0, and overestimations were coded as +1.

For subsequent multivariate analyses, two different total scores, one for the answers in degrees and the other for the answers as percentages, were calculated as the sum of correct answers for each participant, recoding correct answers as 1 and both the over and underestimations as 0 (each total score ranged therefore between 0 and +7).

A multivariate analysis of co-variance (MANCOVA) was then performed to investigate the influence of previous experience, history of incidents, and on-farm occupation on the comprehension of the safety signs, with the total scores for degrees and percentages as dependent variables, on-farm occupation, and involvement in previous incidents as factors and years of experience as a covariate. The statistical analysis was performed with SPSS software v.24 (IBM Corp., Armonk, NY, USA).

## 3. Results

### 3.1. Characteristics of the Participants

The main sociodemographic characteristics of the participants are reported in Table 1. Regarding previous incidents, eight participants reported that they had previously overturned their tractor, whereas three other participants reported other kinds of incidents involving agricultural machinery (i.e., a rear-end collision and slipping from the stepladder).

### 3.2. Comprehension of Safety Signs: Tilt Angles in Degrees vs. Slope Percentages

When considering the answers expressed by all of the participants for the seven levels of slope steepness investigated, a total of 126 answers were collected for angles in degrees and 126 answers for slope percentages. The angles expressed in degrees that yielded the highest correct interpretations were the 5° and 45° angles, whereas the slope, as expressed as a percentage that yielded the highest number of correct interpretations, was 9% (see Table 2). Overall, the angles in degrees were mainly overestimated, whereas the slope percentages were mainly underestimated. As shown in Table 2, 85 out of 126 answers overestimated the actual tilt angle, whereas only 11 answers reported an underestimation. The opposite trend was found for slopes that were expressed in percentages: seventy-one out of 126 answers underestimated the slope, whereas 19 answers reported an overestimation. Interestingly, no participants gave a correct answer for all seven levels of slope steepness investigated, either as a degree or as a percent.

The MANCOVA showed that on-farm occupation and previous history of incidents had no significant main effect on the answers in degrees and percentages (F (2, 12) = 2.306, *p* = 0.142, and F (2, 12) = 0.026, *p* = 0.974, respectively). However, the analysis showed a significant Previous Incidents x On-farm Occupation effect (F (2, 12) = 8.498, *p* = 0.005): when having been previously involved in an incident, farmers were significantly more accurate in estimating the inclination angles in degrees, moving from a mean total score of 0.000 to 2.250. Years of experience with machinery showed no statistically significant effect (F (2, 12) = 0.696, *p* = 0.518) on the comprehension of safety signs.

## 4. Discussion

Previous research has reported a high rate of tractor rollover incidents in agriculture that are caused by a steep slope [1], and safety signs play a key role in warning users against these hazards [52]. In this study, the comprehension of two different graphical representations of slope steepness, in degrees and percent values, was investigated, while also considering the possible effects of previous experience with machinery, history of incidents, and on-farm occupation. Overall, the number of correct estimations was similar between degrees and percentages; however, incorrect answers tended to overestimate the slope when expressed in degrees and to underestimate the slope when it was expressed in percent values. Being a farmer that was previously exposed to a machinery-related incident significantly enhanced the estimation accuracy for tilt angles expressed in degrees.

Drivers’ accuracy in slope angle estimation is an important component of avoiding vehicle rollovers [14], which is one of the most common and dangerous types of tractor incidents, and the overestimation of a slope expressed in degrees can be considered to be a protective behaviour. In other words, even when operators were not accurate in slope estimation, when it was expressed in degrees, the error was in a protective direction, since they would presumably not operate the machinery on a slope that they think is steeper than it actually is. The present results on the overestimation of slopes expressed in degrees are consistent with those that were reported by Görücü et al. [15] and with other previous studies, in which, when the responses were orally reported, the participants tended to overestimate the investigated angles [50,53,54].

However, contrary to the results that were reported by Tillapaugh et al. [14], where “participants were less able to accurately estimate angle as slope became steeper” (p. 262), our results revealed an accurate and similar rate of correct estimations for both less steep angles (5° and 10°) and the steepest ones (38° and 45°), whereas the lowest rate of correct answers was reported for the intermediate angles (15°, 20°, and 25°). The results that were obtained for these latter values are even more critical if we consider that the user’s manual and warning labels on machinery recommend to not operate the machine above these slope limits [13], since it would threaten the stability of the machine or compromise the efficiency of the braking system.

Contrary to the results of other studies, years of experience in driving agricultural machinery, previous history of incidents, and on-farm occupation did not affect the estimation of slope steepness per se. However, a significant interaction effect between on-farm occupation and a history of incidents with agricultural machinery was found. The analysis showed that farmers who had reported a previous incident with agricultural machinery were more accurate in slope estimations when reporting in degree values. As regards the role that is played by operators’ previous experience of incidents, Chan and Ng [41], found similar results, in which the injury experience during working hours significantly enhanced individual performance regarding the comprehension of safety signs. This result is consistent with those of Caffaro et al. [55], in which participants who survived an incident developed a higher risk perception and more careful behaviour. With regard to this, a possible development of the present research could be to investigate the probability of adopting correct and precautionary driving behaviours in agricultural machinery operators with different exposures to previous incidents. Focused training activities and high engagement in safety training are considered to be useful tools in improving the comprehension of safety communication and safety awareness [56] to reduce fatal injuries. This holds true, not only for operators with lower rates of safety sign comprehension, but also for other operators, since, as reported by Chan and Ng [41], “…even people who perform relatively well in guessing, such as those with safety incident experience, will also benefit from such training [...]” (p. 695).

The issue of the most appropriate method of communication about slopes is worthy of further investigation with a larger sample of operators for more reliable results, when comparing, for instance, expert and novice drivers and/or older and younger operators. An additional future development of the research could also address differences in the estimation of slope steepness between male and female agricultural operators in gender-balanced samples. A participatory approach [57] that is based on the iterative and interactive involvement of users throughout the design process of communication regarding slopes could be adopted to better account for tractor and self-propelled machinery operators’ informative needs and to increase the level of comprehension of safety information regarding slopes, which may help to maximize operators’ preventive behaviours. Future developments of the study could also address the relationship between configuration of, comprehension of, and compliance with safety signs, based on previous evidence regarding the association between signs’ configuration and drivers’ behaviour in reducing vehicle speeds in highway work zones [58,59,60]. The behaviour of agricultural machinery drivers after being exposed to different types of safety signs on critical slopes could be observed using a stability simulator, as was seen in Cavallo et al. [13].

## 5. Conclusions

According to the hierarchy of controls, hazards in the use of machinery should be eliminated or controlled through design and engineering solutions. In the case of residual hazards, such as slope steepness for tractor rollovers, safety signs warn operators, and help them to develop safe behaviours. Thus, they are designed to be easily comprehended by users. However, adopting different methods to visually communicate information regarding slope steepness could cause confusion and reasonably lead operators to perform incorrect behaviours while driving the machines. The present results, despite the small number of participants that are involved in the study, highlight some critical aspects that may deserve further attention in future investigations. Expressing slope steepness in degrees appeared to be more protective for operators when compared to percentages; indeed, the steepness was often overestimated in the case of tilt angles expressed in degrees, whereas slope percentages were mainly underestimated. Even though operators might be expected to be more comfortable with this latter unit of measurement, since road signs report slopes as a percentage grade, this investigation showed that this might not always be the case for agricultural machinery operators, highlighting the importance of assessing target users’ knowledge. Moreover, the significant interaction between having been previously involved in machinery-related incidents and on-farm occupation on safety sign comprehension raises some considerations regarding the possibility of designing targeted training interventions to promote the early detection of hazards and risks. Overall, the findings of the present study indicate that the quality of the interaction between individual variables and processes and sign features should be taken into consideration during the design process of safety signs regarding slopes to ensure that safety communications are more comprehensible and possibly more effective.

## Figures and Tables

**Table 1:** Sociodemographic characteristics of the participants.

Variable		*N* (%)
Gender	Male	17(94.5)
	Female	1 (5.5)
Occupation ^1^	Farmer	7 (38.9)
	Farmworker	8 (44.4)
	Contractor	3 (16.7)
		**Mean (SD)**
Age (years)		38.9 (13.0)
Driving experience (years)		25.0 (13.0)

^1^ Contractor = someone who is temporally hired jointly with specific equipment to perform farm work at a certain price or within a certain timeframe. For the subsequent analysis, this variable was re-categorized and coded as 1 = farmer and 0 = other to make group sizes more comparable.

**Table 2:** Answers reported for each angle investigated (both degrees and percentages).

Tilt Angle	Correct Answers *n* (%)	Overestimation *n* (%)	Underestimation *n* (%)	Min (°)	Max (°)	Slope Percentage	Correct Answers *n* (%)	Overestimation *n* (%)	Underestimation *n* (%)	Min (%)	Max (%)
5°	7 (38.8)	11 (61.1)	0 (0)	5	20	9%	14 (77.7)	1 (5.5)	3 (33.3)	2	15
10°	6 (33.3)	11 (55.5)	1 (5.5)	5	30	18%	5 (27.7)	4 (22.2)	9 (50.0)	5	31
15°	2 (11.1)	15 (77.7)	1 (5.5)	12	45	27%	5 (27.7)	4 (22.2)	9 (50.0)	8	50
20°	1 (5.5)	16 (83.3)	1 (5.5)	15	40	36%	6 (33.3)	2 (11.1)	10 (55.5)	13	50
25°	2 (11.1)	14 (72.2)	2 (11.1)	20	60	47%	2 (11.1)	5 (27.7)	11 (61.1)	15	80
38°	5 (27.7)	10 (50)	3 (16.6)	35	50	78%	2 (11.1)	3 (16.6)	13 (72.2)	20	100
45°	7 (38.8)	8 (44.4)	3 (16.6)	30	70	100%	2 (11.1)	0 (0)	16 (88.8)	35	100
